# Biosynthesis of fosfomycin-loaded CuO nanoparticles: evaluation of antibacterial, antibiofilm properties and molecular docking analysis against biofilm-associated proteins in MDR bacteria

**DOI:** 10.2478/abm-2026-0003

**Published:** 2026-04-30

**Authors:** Zukhra Abbasi, Fehmida Fasim, Sehrish Abbas, Rabia Shafique, Barkat Ali Khan, Amna Nisar, Sultan M. Alshahrani, Bushra Uzair

**Affiliations:** Department of Biological Sciences, International Islamic University Islamabad,Pakistan; The University of Notre Dame Fremantle Campus,Australia; Faculty of Rehabilitation and Allied Health Sciences, Riphah International Islamic University, Islamabad 44600, Pakistan; School of Interdisciplinary Engineering and Sciences (SINES), National University of Science and Technology, Islamabad, Pakistan; Department of Pharmaceutics, Faculty of Pharmacy, Gomal University, D.I. Khan 29050, KPK, Pakistan; Department of Clinical Pharmacy, College of Pharmacy, King Khalid University, Alfaraa, Abha 62223, Saudi Arabia

**Keywords:** antibiofilm, antibiotic resistance, Fos-CuO NPs, in silico study

## Abstract

**Background:**

Infectious diseases caused by antibiotic-resistant bacteria pose a significant challenge in healthcare. The development of new antibiotics, while essential, is often hindered by the complexity, cost, and time involved in the process. An alternative approach gaining traction is the conjugation of existing antibiotics with potent antimicrobial agents to improve their efficacy against resistant pathogens.

**Objective:**

This study aimed to develop environmentally sustainable and cost-effective copper oxide nanoparticles (CuO NPs) synthesized using bioactive compounds extracted from *Curcuma zedoaria*.

**Methods:**

These nanoparticles were subsequently conjugated with fosfomycin. Physicochemical characterization was carried out using XRD, scanning electron microscopy (SEM), FTIR, and UV-Visible spectroscopy. Release was studied using Franz diffusion cell. Antibacterial efficacy of the pure and fosfomycin-conjugated copper oxide nanoparticles (Fos-CuO NPs) was evaluated against multidrug-resistant (MDR) strains of *Staphylococcus aureus*, *Escherichia coli*, and *Pseudomonas aeruginosa* using the disk diffusion method. The minimum inhibitory concentration (MIC) and antibiofilm activity were determined using the microbroth dilution method. Additionally, molecular docking analysis was performed to examine the interaction of Fos-CuO NPs with biofilm-associated proteins (LecA, CdrA, PslA, PslD, GacA, CupA, DipA, PelA, PelB) in *P. aeruginosa*.

**Results:**

The physicochemical analysis confirmed successful CuO NPs synthesis and their conjugation with fosfomycin. XRD results confirmed the crystalline structure of the nanoparticles, while SEM revealed some agglomerated, irregular spherical shapes. Fos-CuO NPs exhibited greater antibacterial activity against MDR *S. aureus* (42 mm), *E. coli* (45 mm), and *P. aeruginosa* (39 mm) compared with pure CuO NPs (39 mm, 27 mm, and 41 mm, respectively). The docking results showed that the fosfomycin-conjugated nanoparticle exhibited the highest binding affinity for the biofilm-associated proteins Lec A and Pel A, with docking scores of −4.4 kcal/mol and −4.9 kcal/mol, respectively, compared with blank CuO NPs, supporting their potential application as a novel antimicrobial strategy.

**Conclusion:**

This research offers significant insights into the green synthesis of fosfomycin-conjugated nanoparticles for addressing the growing challenge of multidrug-resistant bacterial infections.

The dramatic spread of antibiotic resistance is one of the foremost existing challenges to human and animal health worldwide, as well as to food security and sustainable development. The WHO has designated AMR as one of the top 10 global health issues in 2019 [1]. The current rise in “superbugs” ranging from *Carbapenen*-resistant *Enterobacteriaceae* (CRE), Vancomycin-resistant *Enterococcus* (VRE), Methicillin-resistant *S. aureus* (MRSA), multidrug-resistant (MDR), and *P. aeruginosa* has become a reason for concern. Individuals suffering from infections caused by resistant micro-pathogens are at a high risk of mortality and morbidity as current antibiotics are unable to treat them, which shows the severity of the issue. If AMR continues unabated, even the best primary healthcare will become ineffective [2].

The scientific community recommends policies to curb the worrying spread of AMR. As per the data reported by Neill [3], the fatal AMR has already caused 700,000 deaths globally and if it continues at this rate, then by 2050 the death toll will be around 10 million every year. Although, with advancement in the industrialization of antibiotics, diseases have gone from being deadly and untreatable to being curable. However, antibiotic resistance has been a threat to humans and animals at the same time. Relevant statistics show that antimicrobial resistance relates to almost all known natural and synthetic antimicrobial agents, as it is a naturally occurring phenomenon in bacteria. This results in the development of new types of bacteria that are no longer susceptible to the antibiotics currently in use [4]. Efforts are being made to find a cure for drug-resistant pathogenic bacteria by minimizing the use of antibiotics and finding a good alternative for them. However, owing to the high cost and lengthy process of clinical trials, a few drugs have been discovered in past years, and AMR is still difficult to control [5].

Metallic nanoparticles have proven to be useful in various applications, including imaging, sensing, and therapeutics [6]. Recently, metal oxide (MOx) nanoparticles have gained much attention for their use in catalysis, cosmetics, optoelectronic materials, and drug delivery [7]. Inorganic MOx nanoparticles such as CuO, ZnO, SiO_2_, NiO, TiO_2_, and Al_2_O exhibit exceptional antimicrobial properties against a wide spectrum of bacterial species, irrespective of their Gram classification [8].

Physical, chemical, and electrochemical approaches were employed for the synthesis of metal nanoparticles, but they possess certain limitations such as the utilization of toxic chemicals and the use of hazardous waste. Now with the development in nanotechnology, environmentally friendly fabrication methods of nanoparticles are preferred. In recent years, immense research has been carried out on the MOx nanoparticles utilization, as they exhibit distinct properties of small diameter and high surface area to volume ratio. Plant-mediated synthesis is preferred as it produces cost-effective, environmentally friendly, stable nanoparticles. Apart from this, the green synthesis of nanoparticles via plants makes it easy to scale-up for larger production of nanoparticles [9]. Copper oxide nanoparticles (CuO NPs) can be used in superconductors, gas sensors, batteries, and catalysts for wastewater treatment and have potential efficiency in antimicrobial and anticancer applications. Cu and Cu complexes have been used by human beings for centuries for various purposes such as antimicrobial, antifouling agents, water purifiers, and algaecides [10]. *C. zedoaria* extracts are reported to be used in various pharmaceutical activities, including antimicrobial, antiparasitic, antitumor, anti-allergen, anti-inflammatory, and analgesic effects [11].

*C. zedoaria* mediated CuO NPs via the green synthesis approach and conjugation of CuO NPs with a last resort anti-biotic fosfomycin is reported for the first time in this study.

## Methods

### Materials

*C. zedoaria* powder was collected from NARC Islamabad. Copper acetate, fosfomycin antibiotic, and nutrient agar were purchased from Sigma-Aldrich, and all the chemicals used were of high-quality analytical grade.

### Isolation of bacterial strains

Clinical isolates of *E. coli*, *S. aureus*, and *P. aeruginosa* were obtained from urine, blood culture, and pleural fluid samples, respectively, and identified using selective media (MacConkey and EMB agar for *E. coli*; Mannitol Salt Agar for *S. aureus*; Cetrimide agar for *P. aeruginosa*). A total of 9 *E. coli*, 10 *S. aureus*, and 7 *P. aeruginosa* strains were isolated from these samples. Among them, isolates of each species that exhibited multidrug resistance (resistant to ≥3 antibiotic classes, per CLSI guidelines) were selected for further study.

### C. zedoaria powder extract preparation

The extract was prepared by suspending 6 g of *C. zedoaria* plant powder in 100 mL of DI water and kept at 60°C for half an hour with continuous stirring. The solution was filtered via Whatman filter paper to obtain an aqueous extract and stored at 4°C [12].

### Qualitative analysis of phytochemicals

The phytochemical analysis of the *C. zedoaria* powder was performed as described in previous studies [13].

### Green fabrication of CuO NPs

**Figure 1.**
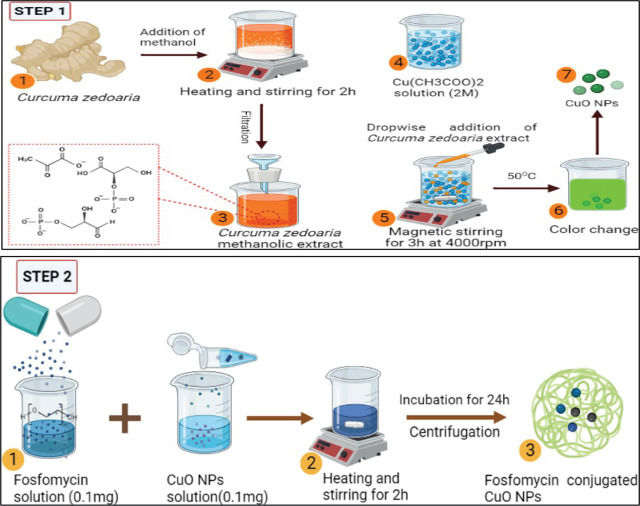
Schematic representation of *C. zedoaria* mediated synthesis of CuO NPs (Step-1); Fos-CuO NPs (Step-2). CuO NPs, copper oxide nanoparticles; Fos-CuO NPs, Fosfomycin conjugated copper oxide nanoparticles.

Fabrication of CuO NPs was performed using the methodology of Veisi and team fellows with some modifications [12]. Plant extract of 25 mL was added to the 2M copper acetate solution and with continued stirring for 3 h at 60°C. Change in color from blue to green was an indication of the synthesis of nanoparticles. CuO NPs were centrifuged to remove unreacted reagents and dried for further use as shown in **Figure 1**.

### Fosfomycin-conjugated copper oxide nanoparticles

Fosfomycin-conjugated copper oxide nanoparticles (Fos-CuO NPs) were prepared by the continuous stirring of CuO NPs and antibiotic solution overnight. The suspension was centrifuged, and the obtained pellet was dried to collect the fosfomycin and CuO NPs complex as shown in **Figure 1**.

### Characterization of CuO NPs

The reduction of Cu^2+^ ion solution by using the methanolic extract of *C. zedoaria* was examined using UV-visible spectroscopy. The nanoparticles powder was resuspended in double-distilled water and subjected to a wavelength scan ranging from 200 nm to 1,100 nm. The Fourier Transform Infrared spectra were recorded for functional group analysis using FTIR Spectrometer in the 400–4,000 cm^−1^ region. XRD was employed to determine the crystalline structure, phase identity, and crystallite size by X—Diffraction model: D8 ADVANCE BRUKER with X—Source Copper (Anode). The morphological analysis of CuO NPs was done by scanning electron microscopy (SEM).

### In-vitro fosfomycin release behavior from biogenic CuO NPs

The release study for the fosfomycin drug from CuO NPs was conducted on a Franz diffusion cell apparatus with an artificial membrane for 12 h at a time interval of 2 h. The percentage of fosfomycin released from the CuO NPs was calculated using the following formula [14]:

Drug release%=Absorbance of sample/Absorbance of standard ×100

### Antibiotic sensitivity profile of isolated strains

The samples were cultured on the respective selective media, such as Cetrimide agar for *P. aeruginosa*, Mannitol Salt Agar for *S. aureus*, and MacConkey agar and Eosin Methylene Blue agar for *E. coli*. A total of 9 strains of *E. coli* were isolated; among these, only one strain exhibited resistance to imipenem, fosfomycin, fluoroquinolones, and cefazolin. Likewise, among 7 isolated *P. aeruginosa*, one was resistant to ceftazidime, gentamicin, and ticarcillin. In addition, among 10 isolated *S. aureus*, only one demonstrated resistance to cephalosporins, doxycycline, and clindamycin. From this collection, only MDR isolates—characterized by resistance to 3 or more antibiotic classes according to CLSI guidelines—were selected for the present study.

### Antibacterial analysis of biogenic CuO NPs and Fos-CuO NPs

The antibacterial potential of green synthesized CuO NPs was checked against MDR *E. coli*, *P. aeruginosa*, and *S. aureus* strains by disk diffusion method [15]. Disks of 5 mm in diameter were immersed in the solutions of CuO NPs and Fos-CuO conjugate. Bacterial lawn was prepared using overnight cultures of all the strains and nanoparticles disks of 5 mm were placed on the prepared lawn. Subsequently, the plates were incubated to calculate the inhibition zone (ZI) after 24 h at 37°C.

### Determination of minimum inhibition concentration and minimal bactericidal concentration

MIC was defined as the lowest nanoparticle concentration at which no visible microbial growth occurred, determined by assessing turbidity both before and after the incubation period. Minimum inhibition concentration of synthesized CuO NPs and Fos-CuO NPs was performed by the methodology of Kanwal et al. [16] with certain modifications against MDR *S. aureus*, *E. coli*, and *P. aeruginosa* bacterial strains using the micro broth dilution method. The McFarland standard (0.5) for bacterial strains was prepared by suspending 2–3 bacterial colonies in normal saline, followed by incubation for 30 min. This was verified using a densitometer at an optical density (OD) of 1.0, and 20 μL of prepared inoculum was transferred to each well. Additionally the synthesized nanoformulations (CuO NPs and Fos-CuO NPs) were diluted in a twofold manner (ranging from 0.01 μg/mL to 1,000 μg/mL), transferred into wells, and incubated for a duration of 24 h. The OD was then measured at 590 nm. Synthesized nanoformulation was tested in triplicate in reference to a tube comprising nutrient broth with bacterial suspension as positive control (PC), and the tube with only broth was considered as negative growth control (NC). Subsequently, minimum bactericidal concentrations (MBC) was determined from the least turbid wells using the spread plate method. The MBC was identified as the lowest nanoparticle concentration that prevented visible growth on the agar surface, corresponding to a ≥99.9% reduction in viable bacterial cells compared with control conditions.

### Determination of fractional inhibition concentration index

The antibacterial effects of fosfomycin and CuO NPs, both individually and in synergistic combination, were evaluated against *P. aeruginosa*, *S. aureus*, and *E. coli* to determine their interactions (whether synergistic, indifferent, or antagonistic) using the fractional inhibitory concentration index (FICI). The fractional inhibitory concentration (FIC) is defined as the minimum inhibitory concentration (MIC) of the antimicrobial agent in combination divided by the MIC of the agent used alone, while the FICI is the sum of the FICs of the 2 antimicrobial agents. The FIC was calculated as the MIC of fosfomycin in combination, divided by the MIC of fosfomycin used independently. The FICI, obtained by the sum of individual FICs, is interpreted as follows: a value of ≤1 indicates synergy, >1–4 signifies indifference, and >4 denotes antagonism.

### Anti-biofilm activity

Antibiofilm assay was assessed using a 96-well microtiter plate method against tested strains. Fos-CuO NPs were diluted to their MIC concentration, further diluted twofold, added to wells, and then incubated for 48 h. ELISA auto reader was used to measure the absorbance at 600 nm. Biofilm inhibition percentage was determined using the following formula [16]:

Biofilm inhibition percentage=OD(control)−OD(treated)/OD(control)×100

### Phenotypic biofilm suppression assay

The biofilm activity of Fos-CuO NPs against *P. aeruginosa* was assessed using a crystal violet assay with animal bone [12]. MIC value of synthesized Fos-CuO NPs was added to 150 mL of sterile nutrient broth containing 20% v/v standardized inoculum of *P. aeruginosa* in a beaker, followed by incubation for 72 h at 37°C. Beaker containing sterile NB was taken as the negative control, whereas the beaker with biofilm-forming *P. aeruginosa* was taken as the PC. After a 72-h incubation period, the bones were washed twice with PBS and stained with 300 μL of CV stain followed by incubation for 30 min. The bones were rinsed twice again with PBS to remove excessive dye and OD was measured to quantify biofilm forma-tion. The percentage of biofilm inhibition was assessed by comparing the OD of untreated control versus Fos-CuO NPs treated tubes. Approximately 10 μL inoculum from Fos-CuO NPs treated and untreated control tubes was spread on agar plates to measure quantitative bacterial counts and biofilm inhibition percentage in Fos-CuO NPs treated and untreated control tubes by standard spread plate assay. The percentage of reduction in biofilm was calculated using the formula [12]:

%Biofilm inhibition=(1−(SA/CA))×100

where SA is the absorbance of sample and CA is the absorbance of PC.

### In silico interaction of biofilm-associated proteins by Fos-CuO NPs

Molecular docking analysis was performed to evaluate the binding score and interactions of fosfomycin, copper (II) oxide, and fosfomycin-conjugated nanoparticles with biofilm-associated proteins of *P. aeruginosa*, including Lec A (PDB: 3ZYH), Pel A (PDB: 5WFT), Pel B (PDB: 5TCB), gacA (Accession: Q51373), pslA (Accession: Q9I1N).

### Preparation of ligands

The molecular structures of fosfomycin and copper (II) oxide were retrieved from ChEMBL and ChemSpider databases, respectively [17, 18]. The structure of the fosfomycin-conjugated nanoparticle was generated using the molecular operating environment (MOE) software based on its simplified molecular input line entry system (SMILES) notation. The SMILES string was obtained through CACTUS Online SMILES Translator [17]. All the structures were further prepared and optimized before docking to ensure conformational stability and compatibility with the molecular docking workflow.

### Preparation of target proteins

The 3D structures of biofilm-associated proteins in *P. aeruginosa*, including Lec A (PDB: 3ZYH), Pel A (PDB: 5TCB), Pel B (PDB: 5WFT), were retrieved from the RCSB Protein Data Bank (www.rcsb.org/). For the biofilm-associated proteins gacA and pslA, which lack experimentally determined crystal structures, the AlphaFold structures were retrieved from the Uniprot database (www.uniprot.org/) under accession numbers Q51373 and Q9I1N8, respectively. All protein structures were prepared using the MOE software, which involved the removal of co-crystallized ligands, addition or deletion of water molecules as appropriate, and charge correction and energy minimization to ensure structural stability before docking.

### Molecular docking

Molecular docking studies were performed using PyRx (an integrated virtual screening tool that employs AutoDock Vina for docking) [18]. A total of 5 biofilm-associated proteins of *P. aeruginosa* and 3 nanoparticle-based ligands were used in this study. The proteins and ligands were converted into PDBQT format using the built-in tools of PyRx. Due to the novelty of our conjugated nanoparticle, no prior knowledge of the binding pocket was available. Therefore, a blind docking strategy was employed by setting the grid box on the entire protein of each target. This ensures the comprehensive exploration of all potential binding sites.

### ADME analysis

SwissADME (http://www.swissadme.ch) was used to validate the drug-likeliness of fosfomycin-conjugated nanoparticle. This tool enables us to check the structural, biochemical, physiochemical properties of our molecules, but also investigate pharmacokinetics (PK) and toxicity.

### Time kill assay

Time kill assay was performed against MDR *S. aureus*, *E. coli*, and *P. aeruginosa* using the method of Rani et al. [19, 20], with certain modifications. Bacterial suspensions for isolated strains were prepared and adjusted to McFarland standards. Solutions of CuO NPs and Fos-CuO NPs were prepared at minimum inhibition concentration (MIC) and added to the aforementioned bacterial suspension. The PC consists of bacterial suspensions without the synthesized nanoparticles solution, while negative control consists solely of broth.

### Statistical analysis

One-way ANOVA, repeated measures (RM) one-way ANOVA, and unpaired t-test was performed using the GraphPad Prism version 10.0.0 for Windows, GraphPad Software, Boston, Massachusetts USA. The mean and standard deviation of the data were estimated.

## Results

### Qualitative analysis of phytochemicals

**Figure 2.**
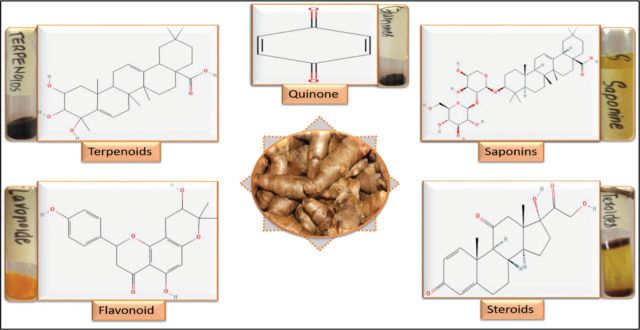
Phytochemical analysis of methanolic extract of *C. zedoaria* powder.

The presence of promising bioactive constituents such as terpenoids, flavonoids, and steroids in *C. zedoaria* rhizome extract was examined via phytochemical screening (**Figure 2**). Phytochemical analysis of the methanolic extract of *C. zedoaria* revealed the presence of reducing agents (terpenoids, flavonoids, and steroids) involved in metal ions reduction. *C. zedoaria* with reducing agents including flavonoids and terpenoids involved in Cu^2+^ ions reduction to CuO. The Cu^2+^ ions were formed by the dissociation of copper acetate into Cu^2+^ and C_2_H_3_O^2−^ ions. Color change from blue to green is the indication of synthesized nanoparticles. The results were consistent with the recent Enicostemma axillare-based synthesis of CuO NPs by Eid and colleagues [21].

### Characterization of biogenic nanoparticles

#### UV–visible absorption spectrometry

**Figure 3.**
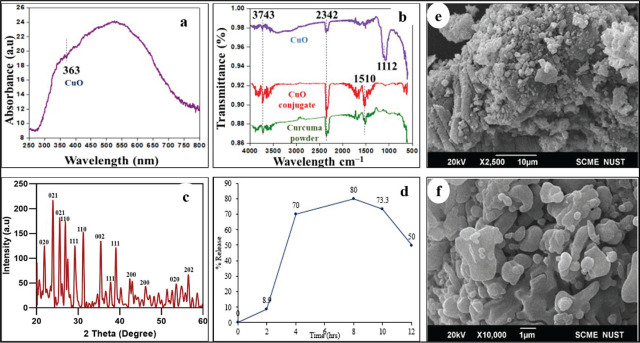
**(A–C)** UV visible spectroscopic and XRD analysis of CuO NPs. **(B)** FTIR analysis of CuO NPs, Fos-CuO conjugate, and *C. zedoaria* powder. **(D)** In vitro release profile of fosfomycin from CuO NPs and **(E,F)** SEM images of CuO NPs at 10 μm and 1 μm. CuO NPs, copper oxide nanoparticles; Fos-CuO, Fosfomycin conjugated copper oxide; SEM, scanning electron microscopy.

The morphological features such as shape, size, and aggregations of synthesized nanoparticles are crucial parameters determining the bioactivity of particles. The color change due to CuO NPs formation was further measured using UV-visible spectrometry (200–1,100 nm) to detect surface plasmon resonance and was observed at 363 nm as shown in **Figure 3A**. The presence of SPR peak at larger wavelengths might be due to factors such as the concentration of metal precursor, size, shape, and aggregations of synthesized nanoparticles [22]. The experimental results are in accordance with the literature as the absorbance wavelength closely matches the results of Saif and his team [23]. Aziz and his coworkers detected an absorbance peak at 346 nm for CuO NPs formation from mint leaf extract [24]. Another literature study reported the promi-nent peak of CuO NPs at 325 nm synthesized by the extracts of orange peel and mint leaf [25].

#### FTIR analysis of CuO NPs

FTIR spectroscopy analysis was done to detect the biomolecules in the methanolic extract of *C. zedoaria*, as well as chemical moieties present in CuO NPs and fosfomycin-CuO conjugate by stretching the frequencies using IR spectra. Furthermore, the interaction between chemical moieties present in *C. zedoaria* extract and their role in precursor salt reduction and converting it into nanoscale in addition to capping and stabilizing of CuO NPs was investigated using FTIR spectroscopy. **Figure 3B** shows the result of FTIR spectra of *C. zedoaria* extract, blank CuO NPs, and Fos-CuO NPs. The *C. zedoaria* extract exhibited several peaks as an indication of extract in complex nature. Within the spectrum, the peak at 3,743 cm^−1^ signifies O-H stretching, alongside a prominent feature at 2,342 cm^−1^, denoting O = C = O stretching. Additionally, the absorption band at 1,510 cm^−1^ can be attributed to hydrocarbon (C-H) groups. Asif et al. [26] reported that the bands at 1,549 cm^−1^, 1,410 cm^−1^ and 1,030 cm^−1^ correspond to hydrocarbon groups (CH, CH3).

Notably, in CuO NPs spectra, the absorption signal shifts toward a shorter wavelength (1,112 cm^−1^) in comparison to the spectra of pure extract (*C. zedoaria*) and Fos-CuO NPs. This absorption band in the range of 1,085–1,150 cm^−1^ stands for C−O bond stretching vibrations in ether. Ghandi and his colleagues observed a sharp band for C−O−C stretching of the acetylene group at 1,087 cm^−1^ that shows resemblance with our experimental result [27]. In another study, conducted by Rohman et al. [28] in 2015, they identified an absorption signal at around 1,100 cm^−1^, which directly corresponds to the stretching vibration of C−O bonds.

Change in peak intensities and formation of new peaks in FTIR spectra of *C. zedoaria* extract, fosfomycin conjugated nanoparticles, and pure CuO NPs confirmed the successful formation of CuO NPs and its loading with fosfomycin [21].

#### X-ray diffraction analysis of CuO NPs

The crystalline nature of the CuO NPs was examined by XRD analysis. **Figure 3C** shows a set of diffraction peaks inferring the crystallinity of CuO NPs. The diffraction peaks of the sample at 2θ of 21.3, 24.4, 26.2, 30.7, 31.8, 33.6, 34.6, 35.2, 38.2, 42.7, 46.5, 53.2, 64.7, and 72.3 corresponds to (020), (021), (021), (110), (111), (110), (002), (111), (200), (200), (020), (202), (113, and (220) planes of crystalline Cu2O, respectively. The phase structure of *C. Zedoaria*-based CuO NPs showed strong diffraction peaks and confirmed the crystalline nature of nanoparticles. Closely related results have been reported in the literature by Tamuly et al. [29].

#### SEM analysis of CuO NPs

Scanning electron microscopic analysis of CuO NPs showed some agglomerated irregular spherical shapes with porous nature as represented in **Figures 3E,F**. Moreover, SEM analysis of synthesized CuO NPs revealed some agglomerated irregular spherical shapes with a porous nature. The findings align with prior research conducted by Mohanarangan and Balakrishnan [30].

#### In-vitro fosfomycin release behavior from biogenic CuO NPs

The in vitro study of fosfomycin from CuO NPs showed burst release at first followed by sustained release, which might be due to the decrease in the concentration of fosfomycin and CuO NPs suspension with time. The maximum release of fosfomycin observed was 80% after 8 h, as demonstrated in **Figure 3D**. Patel et al. [31] reported a significant enhancement in the permeation rate of the drug in a nearly linear fashion when the loading dose was increased.

#### Antibacterial analysis of biogenic nanoparticles

**Figure 4.**
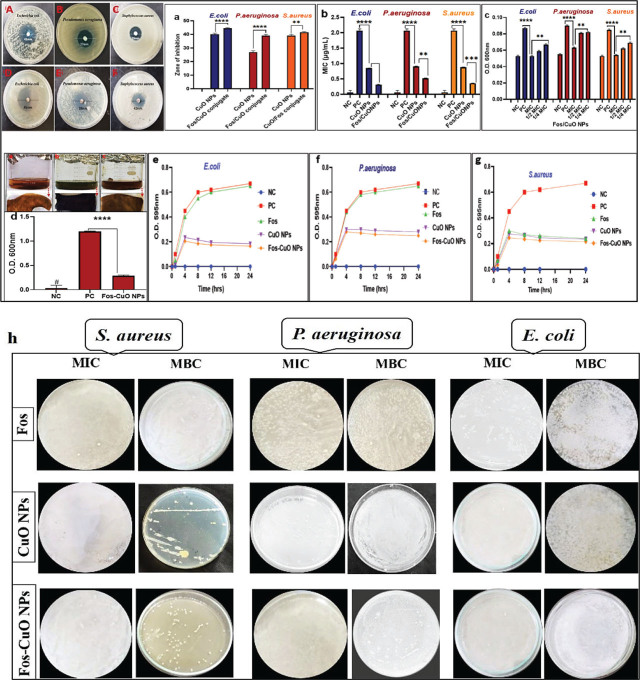
Antibacterial activity of biogenic CuO NPs **(A–C)** and Fos-CuO NPs **(D–F)**. Graphical representation of antibacterial potential **(A)**; MIC assay of CuO NPs and Fos/CuO NPs **(B)**; biofilm inhibition assay of the most effective Fos/CuO conjugate at MIC, ½ MIC and ¼ MIC on microtiter plate **(C)** and teeth sample **(D)** time kill assay graph **(E–G)** and bactericidal activity (MIC: MBC), based on CFU enumeration **(H)** of Fos, CuO NPs and Fos-CuO NPs against *E. coli*, *S. aureus*, and *P. auroginosa*. CuO NPs, copper oxide nanoparticles; Fos-CuO NPs, fosfomycin conjugated copper oxide nanoparticles; MBC, minimum bactericidal concentrations; MIC, minimum inhibition concentration; NC, negative control; PC, positive control.

Antibacterial analysis of CuO NPs and Fos-CuO NPs was checked against 3 MDR bacterial strains, that is, *E. coli*, *S. aureus*, and *P. aeruginosa* using the disk diffusion method as shown in **Figures 4A,F**. Fosfomycin–CuO conjugate exhibited the highest zones of inhibition as compared with CuO NPs. Graphical representation of antimicrobial potential against isolated bacterial strains is presented in **Figure 4A**. In the present study, *C. zedoaria*-mediated CuO NPs and Fos-conjugated CuO NPs were found more effective against Gram-positive bacteria than those of Gram-negative bacteria, which might be due to the presence of additional outer membrane on Gram-negative bacteria [20].

#### MIC, MBC, and FICI of biogenic nanoparticles

MIC of fosfomycin alone was 32 mg/mL for *S. aureus*, 56 mg/mL for *P. aeruginosa* and 50 mg/mL for *E. coli*. The highest degree of cell lysis was observed against *E. coli* only at an MIC value of 0.86 ± 0.5 μg/mL for CuO NPs. Conversely, the synthesized Fos-CuO NPs demonstrated a significant synergistic effect against *E. coli* at an MIC value of 0.32 ± 0.5 μg/mL. CuO NPs exhibited an MIC range of 0.91–0.5 g/mL against *P. aeruginosa*, and 0.89 ± 0.5 μg/mL against *S. aureus*. Meanwhile, the MIC values of Fos-CuO NPs were 0.53 ± 0.5 μg/mL for *P. aeruginosa* and 0.37 ± 0.5 μg/mL for *S. aureus*. Additionally, the MBC of CuO NPs were determined to be 1.78 ± 0.5 μg/mL for *E. coli*, 1.90 ± 0.5 μg/mL for *P. aeruginosa*, and 2.07 ± 0.5 μg/mL for *S. aureus*. The MBC for Fos-CuO NPs was found to be 0.68 ± 0.5 μg/mL against *E. coli*, 1.09 ± 0.5 μg/mL against *P. aeruginosa*, and 0.79 ± 0.5 μg/mL against *S. aureus* (**Figure 4H)**. Overall, the synthesized CuO NPs exhibited enhanced antibacterial activity (*P* < 0.05) against all tested strains following conjugation with fosfomycin, as demonstrated by unpaired t-test analysis (**Table 1**). Using the MIC values of fosfomycin, CuO NPs, and fosfomycin–CuO NPs, the FICI values were determined to be 0.372 for *E. coli*, 0.416 for *S. aureus*, and 0.582–1.060 for *P. aeruginosa* (depending on the MIC range of CuO alone). According to accepted interpretive criteria (FICI ≤ 1, synergy; >1–4, indifference; >4, antagonism), the conjugate displayed synergistic interactions against *E. coli* and *S. aureus*, while an additive effect was observed against *P. aeruginosa*. Interestingly, no evidence of antagonism was observed in any of the tested MDR strains (**Table 2**). The findings are coherent with a study by Kanwal et al. [16] in which they found that carbon-dots decorated CaCO3 (CD-CCNC) nanocarrier conjugated with antibiotic “levofloxacin” had enhanced antibacterial ability.

**Table 1.** Minimum inhibition concentration and MBC of Fosfomycin, CuO NPs, and Fos-CuO NPs

**Test microorganism**	**Fos**		**CuO NPs**		**Fos-CuO NPs**	
		
	**MIC (mg/mL)**	**MBC (mg/mL)**	**MIC (μg/mL)**	**MBC (μg/mL)**	**MIC (μg/mL)**	**MBC (μg/mL)**
*E. coli*	50 ± 0.5	65 ± 0.5	0.86 ± 0.5	1.72 ± 0.5	0.32 ± 0.5	0.64 ± 0.5
*P. aeruginosa*	56 ± 0.5	70 ± 0.5	0.91 ± 0.5	1.82 ± 0.5	0.53 ± 0.5	1.06 ± 0.5
*S. aureus*	32 ± 0.5	46 ± 0.5	0.89 ± 0.5	1.78 ± 0.5	0.37 ± 0.5	0.74 ± 0.5

CuO NPs, copper oxide nanoparticles; Fos-CuO NPs, fosfomycin conjugated copper oxide nanoparticles; MBC, minimum bactericidal concentrations.

**Table 2.** Fractional inhibition concentration of Fos-CuO NPs

**Test microorganism**	**Fos MIC (μg/mL)**	**CuO MIC (μg/mL)**	**Fos-CuO MIC (μg/mL)**	**FICI**	**Interpretation**
*E. coli*	50,000 ± 0.5	0.86 ± 0.5	0.32 ± 0.5	0.37	Synergy
*P. aeruginosa*	56,000 ± 0.5	0.91 ± 0.5	0.53 ± 0.5	0.58	Additive (borderline synergy)
*S. aureus*	32,000 ± 0.5	0.89 ± 0.5	0.37 ± 0.5	0.42	Synergy

Fos-CuO NPs, fosfomycin conjugated copper oxide nanoparticles; MBC, minimum bactericidal concentrations; FICI, fractional inhibitory concentration index.

#### Quantitative biofilm inhibition assay

Fos-CuO showed prominent antibiofilm potential against MDR *E. coli*, *P. Aeruginosa*, and *S. aureus* strains at MIC value (**Figure 4C**). Biofilm disruption percentages of Fos-CuO NPs at MIC were 39% for *E. coli*, 36% for *S. aureus*, and 30% for *P. aeruginosa*. However, the percentage antibiofilm potential decreased to 32% for *E. coli*, 27% for *S. aureus*, and 10% for *P. aeruginosa* at ½ MIC. This percentage was further reduced at ¼ MIC, that is, 22% for *E. coli*, 27% for *S. aureus*, and 10% for *P. aeruginosa*. Uzair et al. [32] also reported a decrease in biofilm disruption percentage at ½ MIC and ¼ MIC.

#### Phenotypic biofilm suppression assay

The antibiofilm effect of synthesized Fos-CuO NPs on *P. aeruginosa* on teeth is shown in **Figure 4D**. The results indicate that the synthesized nanoparticles significantly inhibited biofilm formation (*P* < 0.0001) compared with the control at MIC value of 0.53 ± 0.5 μg/mL. This inhibition is attributed to the interaction between Fos-CuO NPs and the anionic components of the bacterial surface [33]. Treated nanoparticles showed a 75% reduction in biofilm in comparison to untreated and the results align with the study by Tawre et al. [34], where 76.60% biofilm disruption was observed at a concentration of 8 μg/mL against *P. aeruginosa*.

#### Time kill assay of Fos/CuO NPs

Time kill assays revealed that *C. zedoaria*-synthesized nanoparticles significantly reduced bacterial growth over 24 h compared with free fosfomycin (*P* < 0001). Pure CuO NPs and fosfomycin-conjugated formulations achieved early bactericidal activity within 2 h, as evidenced by reduced OD, and demonstrated greater efficacy than free fosfomycin. The OD of the PC increased over time, whereas that of the negative control remained unchanged for 24 h. The synthesized nanomaterials exhibited enhanced activity against Gram-positive *E. coli* and *S. aureus* relative to Gram-negative *P. aeruginosa* (**Figure 4E**).

**Figure 5.**
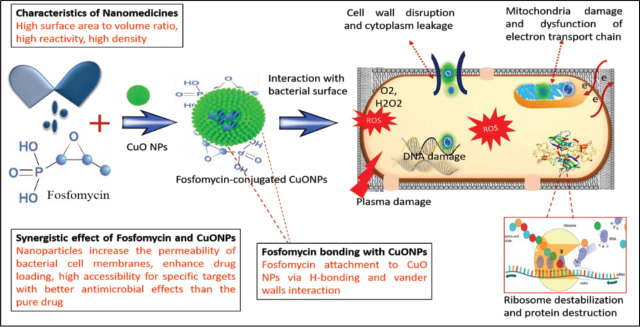
Hypothetical antibacterial mechanism of Fos-CuO NPs. Possible mechanisms include ROS generation, leading to dysfunction of the electron transport chain, and disruption of cell membrane, protein, and DNA. Fos-CuO NPs, fosfomycin conjugated copper oxide nanoparticles.

The antimicrobial potential against all tested strains was observed to be larger after the conjugation of CuO with fosfomycin confirming the successful drug loading. It could be proposed that CuO NPs engage in electrostatic interaction with the outer plasma membrane of bacterial cells and disrupt it due to the production of ROS resulting in cell death. Nanoparticles have less surface area to volume ratio as they have a small size, and can easily bind to the bacterial cell wall which would destroy the cell [35]. Moreover, the released copper ions and fosfomycin cause mitochondria damage, dysfunction of electron transport change, ribosome destabilization, and protein denaturation. Possible antimicrobial mechanisms of synthesized nanoparticles is shown in **Figure 5**.

### In silico interaction of biofilm-associated proteins by Fos-CuO NPs

#### Protein–ligand interactions

The molecular docking analysis was performed to evaluate the binding score and interactions of fosfomycin, copper (II) oxide, and fosfomycin-conjugated nanoparticles with biofilm-associated proteins of *P. aeruginosa*, including Lec A (PDB: 3ZYH), Pel A (PDB: 5WFT), Pel B (PDB: 5TCB), gacA (Accession: Q51373), and pslA (Accession: Q9I1N). The docking scores are summarized in **Table 3**. The docking results showed that the fosfomycin-conjugated nanoparticle exhibited the highest binding affinity for the biofilm-associated proteins Lec A and Pel A, with docking scores of −4.4 kcal/mol and −4.9 kcal/mol, respectively. Both fosfomycin and fosfomycin-conjugated nanoparticle showed the same binding affinity score of −3.7 kcal/mol with Pel B. For gacA and psl A, Fosfomycin alone achieved the highest binding affinities, with docking scores of −4.0 kcal/mol and −4.7 kcal/mol, while fosfomycin-conjugated nanoparticle ranked second for these targets. By contrast, copper (II) oxide (CuO) consistently exhibited the lowest score across all the target proteins, indicating limited interaction potential compared with fosfomycin and its conjugated form.

**Table 3.** The molecular docking scores between ligands and biofilm-associated proteins in *P. aeruginosa*

**Protein**	**Ligands**	**Binding affinity (kcal/mol)**
Lec A	CuO	−2.6
	Fosfomycin	−4.1
	Fosfomycin-conjugated	−4.4
Pel A	CuO	−2.5
	Fosfomycin	−4
	Fosfomycin-conjugated	−4.9
Pel B	CuO	−2.5
	Fosfomycin	−3.7
	Fosfomycin-conjugated	−3.7
gacA	CuO	−2.6
	Fosfomycin	−4
	Fosfomycin-conjugated	−3.9
pslA	CuO	−2.7
	Fosfomycin	−4.7
	Fosfomycin-conjugated	−3.8

#### Interaction analysis of docked complexes

**Figure 6.**
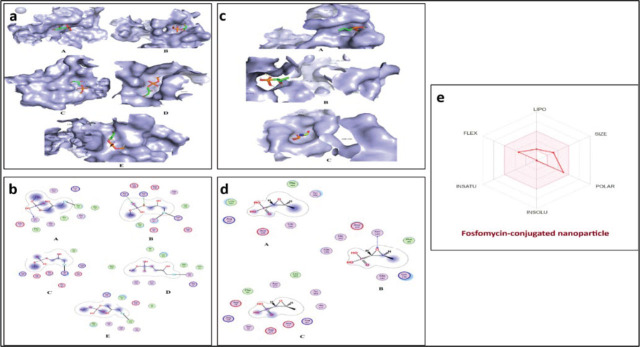
The 3D interaction **(A)** and 2D interaction **(B)** between biofilm-associated proteins and Fos-CuO NPs. **(A)**, Lec A (PDB: 3ZYH); **(B)**, Pel A (PDB: 5TCB); **(C)** Pel B (PDB: 5WFT); **(D)**, gacA (Accession: Q51373); **(E)**, pslA (Accession: Q9I1N). The 3D interaction **(C)** and 2D interaction **(D)** between biofilm-associated proteins and Fosfomycin. **(A)**, Pel B (PDB: 5WFT); **(B)**, gacA (Accession: Q51373); **(C)**, pslA (Accession: Q9I1N) and 3D ADME Radar **(E)** for the graphical representation of Fosfomycin-conjugated nanoparticle for lipophilicity, flexibility, instauration, insolubility, size, and polarity. The pink region of the radar indicates an ideal range of bioavailability to a drug. ADME, absorption, distribution, metabolism, and excretion; CuO NPs, copper oxide nanoparticles; Fos-CuO NPs, fosfomycin conjugated copper oxide nanoparticles.

For each protein–ligand complex, the binding poses were evaluated based on both docking scores and the number of molecular interactions. The pose with the good binding affinity and number of interactions was selected for further analysis. This approach ensures that the ligand fits well energetically within the binding site but also forms key interactions critical for stable binding and potential biological activity. The visual representation of the interaction between fosfomycin-conjugated nanoparticle and biofilm-associated proteins is shown in **Figures 6A–D**, which display the 3D binding poses and 2D interactions, while **Table 4** shows the interacting residues of the biofilm-associated proteins. Additionally, **Figures 5C–D** illustrate the binding poses and interactions of fosfomycin with 3 target proteins (PelB, gacA, and pslA), where it exhibits the highest binding affinity.

**Table 4.** Interacting residues between fosfomycin-conjugated nanoparticle and biofilm-associated proteins in *P. aeruginosa*

**Fosfomycin-conjugated nanoparticle**	**Protein**	**Interacting residues**
	Lec A	Ser111, Trp33
	Pel A	Arg267, Arg271, Gln67, Ser51
	Pel B	Thr387
	gacA	Glu64
	pslA	Leu 150

#### ADME analysis using Swiss ADME

The pharmacokinetic properties of the fosfomycin-conjugated nanoparticle were evaluated using Swiss ADME to predict its absorption, distribution, metabolism, and excretion (ADME) profile. The compound exhibited 5 rotatable bonds, suggesting a flexible structure that could facilitate target binding. The molar refractivity was calculated at 35.76, suggesting favorable molecular polarizability. The compound was predicted to be water-soluble, which is good for formulation and bioavailability. The PK profile indicated high gastrointestinal (GI) absorption but no blood–brain barrier (BBB) permeability, suggesting a selective distribution profile suitable for non-CNS targets. The bioavailability score was estimated at 0.55, reflecting moderate oral bioavailability potential. Drug-likeness assessment showed full compliance with Lipinski's Rules of 5 with zero violations, highlighting the compound's suitability for oral drug development (**Figure 6E**).

## Conclusions

In this study, an economical and eco-friendly green synthesis of CuO NPs was achieved successfully by using the powder extract of *C. zedoaria*. The synthesized CuO NPs showed enhanced bactericidal potential against clinically isolated MDR *E. coli*, *P. aeruginosa*, and *S. aureus* bacterial strains when conjugated with fosfomycin. Thus, it is assumed that this strategy will help to improve the efficacy of multiple antibiotics to fight against drug-resistant bacteria. This conjugation could be used to save fosfomycin as a last-line antibiotic in an era of antibiotic resistance. Moreover, molecular docking analysis indicated highest binding affinity for the biofilm-associated proteins Lec A and Pel A, with docking scores of −4.4 kcal/mol and −4.9 kcal/mol, respectively, compared with pure fosfomycin and CuO NPs. The bioavailability score was estimated at 0.55, reflecting moderate oral bioavailability potential. Drug-likeness assessment showed full compliance with Lipinski's Rules of 5 with zero violations, highlighting the compound's suitability for oral drug development. In conclusion, further experimental and clinical studies are needed to authenticate and strengthen the findings of the current study.
